# Lactate formation from fructose or C1 compounds in the acetogen *Acetobacterium woodii* by metabolic engineering

**DOI:** 10.1007/s00253-023-12637-7

**Published:** 2023-07-07

**Authors:** Jimyung Moon, Lara M. Waschinger, Volker Müller

**Affiliations:** Department of Molecular Microbiology & Bioenergetics, Institute of Molecular Biosciences, Johann Wolfgang Goethe University, Max-von-Laue-Str. 9, D-60438 Frankfurt, Germany

**Keywords:** CO_2_-based bioeconomy, C1 compounds, Lactate, Lactogenesis, Metabolic engineering

## Abstract

**Abstract:**

Anaerobic, acetogenic bacteria are promising biocatalysts for a sustainable bioeconomy since they capture and convert carbon dioxide to acetic acid. Hydrogen is an intermediate in acetate formation from organic as well as C1 substrates. Here, we analyzed mutants of the model acetogen *Acetobacterium woodii* in which either one of the two hydrogenases or both together were genetically deleted. In resting cells of the double mutant, hydrogen formation from fructose was completely abolished and carbon was redirected largely to lactate. The lactate/fructose and lactate/acetate ratios were 1.24 and 2.76, respectively. We then tested for lactate formation from methyl groups (derived from glycine betaine) and carbon monoxide. Indeed, also under these conditions lactate and acetate were formed in equimolar amounts with a lactate/acetate ratio of 1.13. When the electron-bifurcating lactate dehydrogenase/ETF complex was genetically deleted, lactate formation was completely abolished. These experiments demonstrate the capability of *A. woodii* to produce lactate from fructose but also from promising C1 substrates, methyl groups and carbon monoxide. This adds an important milestone towards generation of a value chain leading from CO_2_ to value-added compounds.

**Key points:**

• *Resting cells of the ΔhydBA/hdcr mutant of Acetobacterium woodii produced lactate from fructose or methyl groups + CO*

• *Lactate formation from methyl groups + CO was completely abolished after deletion of lctBCD*

• *Metabolic engineering of a homoacetogen to lactate formation gives a potential for industrial applications*

## Introduction

Acetogenic bacteria are a group of strictly anaerobic bacteria that oxidize one mol of hexoses such as fructose to three mol of acetate, a metabolic trait known as homoacetogenesis (Fontaine et al. 1942). Fructose is oxidized *via* the Embden-Meyerhof-Parnas pathway to four electrons and two mol of pyruvate which are further oxidized to two mol of acetyl-CoA, CO_2_ and four more electrons (Ragsdale 2003). Acetate formation yields 4 mol of ATP per hexose, the highest amount of ATP that can be obtained by fermentation (Müller 2008; Müller and Frerichs 2013). This is only possible by disposing the electrons in a special pathway for CO_2_ reduction to acetate, the Wood-Ljungdahl pathway (WLP) in which two CO_2_ are reduced by eight electrons to acetate (Müller 2003; Wood and Ljungdahl 1991). The WLP is not only an electron sink for fructose oxidation, but also allows acetogens to grow on H_2_ + CO_2_ (Schuchmann and Müller 2014; Wood et al. 1986) or other C1 compounds such as formate (Moon et al. 2021) or methanol (Balk et al. 2003; Kremp and Müller 2021; Kremp et al. 2018; van der Meijden et al. 1984). CO_2_ is reduced in two branches. In the methyl branch, one CO_2_ is first reduced to formate by a formate dehydrogenase, or more specific, by a hydrogen-dependent CO_2_ reductase in the model acetogen *Acetobacterium woodii* (Schuchmann and Müller 2013). Formate is then bound in an ATP-dependent reaction to the C1 carrier tetrahydrofolate (THF) (Himes and Harmony 1973; Lovell et al. 1988), yielding formyl-THF from which water is eliminated and the resulting methenyl-THF is reduced *via* methylene- to methyl-THF (Bertsch et al. 2015; Ragsdale and Ljungdahl 1984). In the second branch, CO_2_ is reduced to CO which is then bound to the key enzyme of the pathway, CO dehydrogenase/acetyl-CoA synthase (CODH/ACS) and combined with the methyl group and CoA to acetyl-CoA (Ragsdale 2008). The substrates formate (Moon et al. 2021) and carbon monoxide (Diekert and Thauer 1978; Diender et al. 2015; Genthner and Bryant 1982; Weghoff and Müller 2016) are intermediates of the pathway and methyl groups from, for example, methanol or glycine betaine, enter the pathway by a methyltransferase system yielding methyl-THF (Kremp and Müller 2021; Kremp et al. 2018; Lechtenfeld et al. 2018).

Acetogenic bacteria have gained much interest in recent years since they capture the greenhouse gas CO_2_ and reduce it to acetate. This small chain fatty acid has limited application *per se*, but acetate may substitute glucose in the long run to a sustainable bioeconomy as feedstock for the production of not only biofuels but also all the other products that are currently produced from sugars by, for example, *Escherichia coli*, *Corynebacterium glutamicum* or yeasts (Förster and Gescher 2014; Ingram et al. 1987; Inui et al. 2004a, b; Jojima et al. 2015a, b; Lim et al. 2018; Mohd Azhar et al. 2017). In addition to acetate, some acetogens can produce ethanol from C1 compounds such as CO_2_ and CO and this process is already used on an industrial scale (Liew et al. 2017, 2022; Mock et al. 2015). Higher carbon compounds are rarely produced and generally not from C1 compounds. A C1 substrate of interest is methanol which is also used by acetogens as carbon and energy source (Kremp and Müller 2021; Kremp et al. 2018; van der Meijden et al. 1984). Methanol is already produced from CO_2_ chemically on an industrial level and the use of methanol as a feedstock circumvents all the problems inherent to gas fermentation.

Recently, we discovered a novel metabolic trait in *A. woodii*, mixed acid fermentation of fructose (Moon et al. 2023a). A mutant in which the central enzyme of the WLP, the methylene-tetrahydrofolate reductase was genetically deleted, was able to grow on fructose. But acetate was not the only product; in addition molecular hydrogen, formate, ethanol and lactate were produced as end products (Moon et al. 2023a). This finding offered the possibility to engineer strains that convert fructose or even C1 compounds to reduced end products such as ethanol or lactate. Production of lactate is of great interest since it is widely used in food, pharma- and cosmetic industries as well as serves as the precursor of a biologically degradable plastic, poly lactic acid (PLA) (Ahmad et al. 2022). Here, we have chosen lactate as a target and generated a strain of *A. woodii* that performs heterolactate fermentation from fructose or from methyl groups plus carbon monoxide.

## Materials and methods

### Strains and cultivation

*A. woodii* wild type (DSM1030) was obtained from the Deutsche Sammlung von Mikroorganismen und Zellkulturen (DSMZ; Braunschweig, Germany). The ∆*pyrE* strain was described before (Wiechmann et al. 2020). The *hdcr* deletion mutant ∆*hdcr* and the double mutant ∆*hydBA/hdcr* were described recently (Moon et al. 2023b). The triple mutant ∆*hydBA/hdcr/lctBCD* in which the genes encoding the lactate dehydrogenase were deleted in addition was generated in this study. All strains were routinely cultivated under anoxic conditions at 30 °C in bicarbonate-buffered complex medium as described before (Heise et al. 1989). As substrates for growth, 60 mM fructose + 100 mM formate, or 50 mM glycine betaine + 10% CO were used. Growth was monitored by determining the optical density at 600 nm (OD_600_).

### Generation of *A. woodii ΔhydBA/hdcr/lctBCD* mutant

To generate the Δ*hydBA/hdcr/lctBCD* triple mutant, the plasmid pMTL84151_LW_dlct was constructed in *E. coli* HB101 (Promega, Madison, WI, USA) and transformed into the *A. woodii* Δ*hydBA/hdcr* strain (Moon et al. 2023b), as described previously (Westphal et al. 2018). The plasmid pMTL84151_LW_dlct originated from pMTL84151 (Heap et al. 2009) but lacks a Gram-positive replicon. In pMTL84151_LW_dlct, 1000 bp of upstream flanking regions (UFR) of *lctB* (Awo_c08710) and 1000 bp of downstream flanking regions (DFR) of *lctD* (Awo_c08730) were cloned into the multiple cloning sites to delete the *lctBCD* genes by homologous recombination. The plasmid also has a *catP* marker from *Clostridium perfringens* coding for chloramphenicol/thiamphenicol resistance (Werner et al. 1977) and a heterologous *pyrE* gene from *Eubacterium limosum* (Wiechmann et al. 2020) as a counter selectable marker. The first selection was carried out on an agar plate with complex medium containing 20 mM fructose + 50 mM formate and 30 ng/µl thiamphenicol after transformation of pMTL84151_LW_dlct into the *A. woodii* Δ*hydBA/hdcr* strain by electroporation (625 V, 25 µF, 600 Ω, in 1 mm cuvettes). The second selection for disintegration was performed on an agar plate with minimal medium (Westphal et al. 2018) containing 20 mM fructose + 50 mM formate, 1 µg/ml uracil and 1 mg/ml 5-fluoroortate (5-FOA). The deleted region was analyzed by PCR with primers binding upstream of UFR and downstream of DFR: aus_lct_for (5′-CAGGCAATGTTTTTTAATGTCAGGA-3′) and aus_lct_rev (5′-ATAACTTTTGCCAAAGCCACAAT-3′). Consequently, PCR experiments were performed to verify the purity of the mutant, with primers binding in the *lctD* gene: in_lct_for (5′-GGTAATATCAGTACGAATGCCGG-3′) and in_lct_rev (5′- GAATCGCCTTGGATTTAATAATCTTCG-3′). Subsequently, the sequence of the deleted region of the mutant was verified by DNA sequencing (Sanger et al. 1977).

### Preparation of resting cells

Cells were cultivated either on 60 mM fructose + 100 mM formate or 50 mM glycine betaine + 10% CO in 1 l bicarbonate-buffered complex medium to the late exponential growth phase (on 60 mM fructose + 100 mM formate, OD_600_ of 1.5; on 50 mM glycine betaine + 10% CO, OD_600_ of 0.7). Cells were harvested by centrifugation (Avanti J-25 and JA-10 Fixed-Angle Rotor; Beckman Coulter, Brea, CA, United States) at 8,000 rpm and 4 °C for 10 min, washed with 30 ml of buffer containing 50 mM imidazole (pH 7.0), 20 mM KCl, 20 mM MgSO_4_, 4 mM DTE and 4 µM resazurin and pelleted by centrifugation at 8,500 rpm and 4 °C for 10 min (Avanti J-25 and JA-25.50 Fixed-Angle Rotor; Beckman Coulter, Brea, CA, United States). Subsequently, the pellets were resuspended in 5 ml imidazole buffer and transferred to 16-ml Hungate tubes. All steps were performed under strictly anoxic conditions in an anoxic chamber (Coy Laboratory Products, Grass Lake, MI, United States) filled with N_2_/H_2_ (96–98%/2–4%; v/v). To get rid of residual H_2_ from the anoxic chamber, the gas phase of the cell suspensions was changed to 100% N_2_. The total protein concentration of the cell suspensions was measured as described before (Schmidt et al. 1963).

### Cell suspension experiments

For fructose fermentation, the cells were resuspended in 20 ml of bicarbonate-containing imidazole buffer (50 mM imidazole, 20 mM KCl, 20 mM NaCl, 20 mM MgSO_4_, 60 mM KHCO_3_, 4 mM DTE, 4 µM resazurin, pH 7.0) in 120-ml serum flasks under a N_2_/CO_2_ atmosphere (80:20, v/v) to a final protein concentration of 2 mg/ml. As substrate, 60 mM fructose was added. For glycine betaine + CO fermentation, resting cells were prepared in 10 ml of bicarbonate-containing imidazole buffer under a N_2_/CO_2_/CO atmosphere (2 bar, 72:18:10, v/v/v) to a final protein concentration of 1 mg/ml. For the experiment under bicarbonate-depleted conditions, bicarbonate-depleted buffer (50 mM imidazole, 20 mM KCl, 20 mM NaCl, 20 mM MgSO_4_, 4 mM DTE, 4 µM resazurin, pH 7.0) was used and the gas phase was replaced to a N_2_/CO atmosphere (2 bar, 90:10, v/v). For the experiments under Na^+^-depleted conditions, Na^+^-depleted buffer (50 mM imidazole, 20 mM KCl, 20 mM MgSO_4_, 60 mM KHCO_3_, 4 mM DTE, 4 µM resazurin, pH 7.0) was used and the contaminating Na^+^ concentration in the buffer was determined with an Orion 84–111 ROSS sodium electrode (Thermo Electron, Witchford, UK) according to the supplier's instructions. As substrate, 50 mM glycine betaine was added to the resting cells. The resting cells were pre-incubated at 30 °C in a water bath with shaking (150 rpm) and the experiments were started by adding the substrate(s). During the experiments, 1-ml samples were routinely taken for metabolite analyses.

### Metabolite analyses

The concentrations of fructose, formate, acetate, and lactate were determined by high-performance liquid chromatography as described previously (Moon et al. 2019). H_2_ or ethanol were analyzed by gas chromatography (Trifunović et al. 2016; Weghoff and Müller 2016).

### Gene expression analyses

The ∆*pyrE*, ∆*hdcr*, ∆*hydBA/hdcr* mutants grown on 50 mM glycine betaine under a N_2_/CO_2_/CO atmosphere (72:18:10, v/v/v) in bicarbonate-buffered complex media were harvested in the exponential growth phase. Preparation of RNA and cDNA was performed as described before (Dönig and Müller 2018). Transcript levels of the *lctB*, *lctC*, and *lctD* genes were analyzed with real-time qPCR in a Rotor Gene RG-3000 qPCR cycler (Corbett Research, Cambridge, UK) using Maxima SYBR Green qPCR Master Mix (Thermo Fisher Scientific, Waltham, MA, USA) following the supplier's instructions. The housekeeping gene *gyrA* (Awo_c00060) was used as reference and the relative gene expression levels were calculated using the 2^−ΔΔCt^ method (Livak and Schmittgen 2001). For the amplification, following primers were used: qlctB_for (5′-GCGCTGATGAGGGTTGTTTA-3′) and qlctB_rev (5′-TCACCCAATCGTTTGGTG-3′) for *lctB*, qlctC_for (5′-GTCGATCATATTGAAGGCCAGAT-3′) and qlctC_rev (5′-ACAAGGCATAAACCGGATGT-3′) for *lctC*, and qlctD_for (5′-GATTCCAACGGCGATTGAAT-3′) and qlctD_rev (5′-TATAAGCGTTGCTACTGGAGTC-3′) for *lctD*.

## Results

### Strain design

**Fig. 1:**
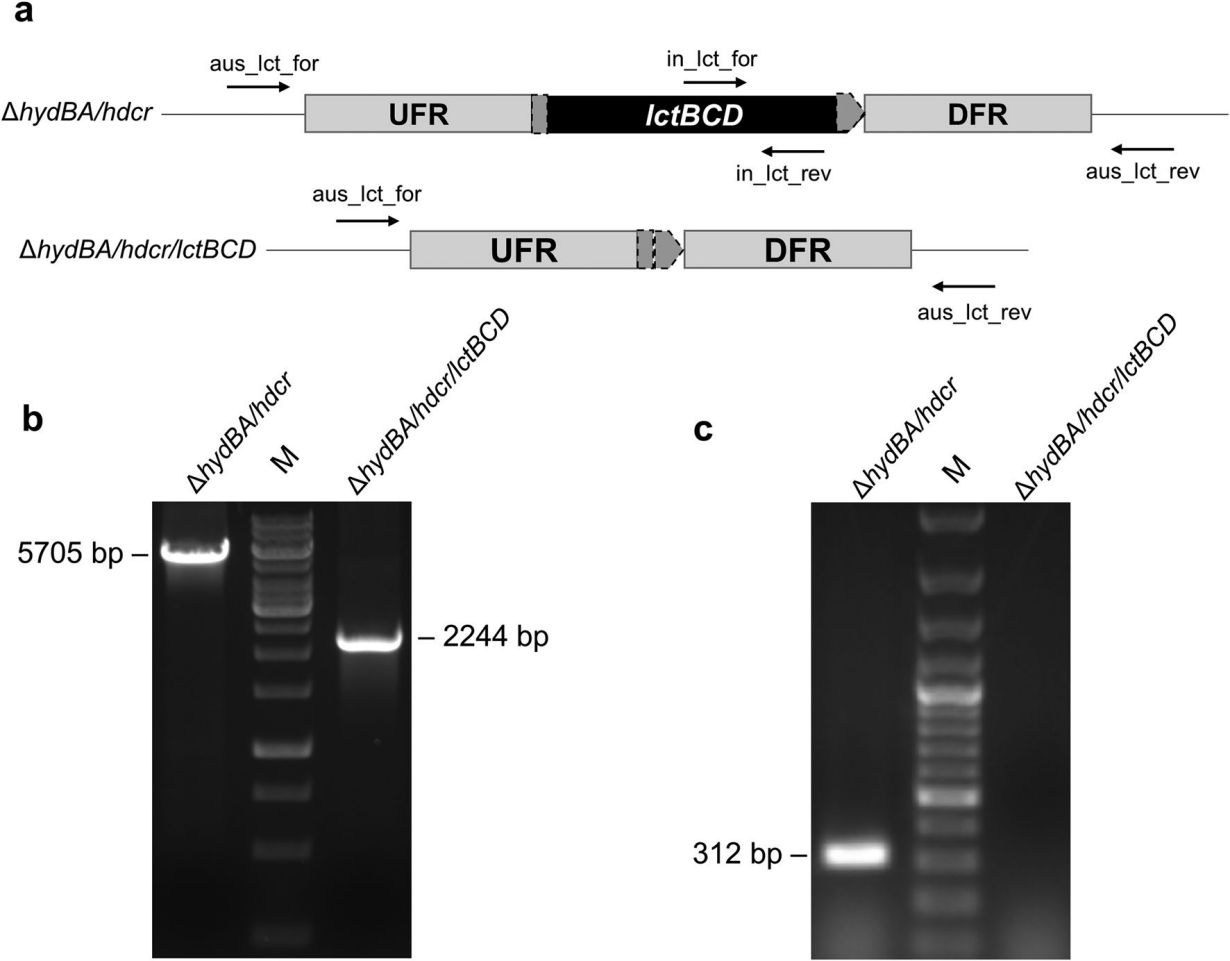
Deletion of the *lctBCD* genes in the chromosome of the Δ*hydBA/hdcr* mutant. (**a**) Genetic organization after deletion of the *lctBCD* genes using plasmid pMTL_LW_dlct. Only 3 bp of the *lctB* gene and 3 bp of the *lctD* gene remained in the Δ*hydB/hdcr/lctBCD* mutant. Genotypic analyses of the Δ*hydBA/hdcr/lctBCD* mutant were carried out by colony PCR with primers binding outside the deleted region (**b**) (aus_lct_for and aus_lct_rev) or inside (**c**) (in_lct_for and in_lct_rev)

There are two hydrogenases encoded in the genome of *A. woodii*, the HydA2 subunit of the HDCR and the electron-bifurcating HydABC hydrogenase (Poehlein et al. 2012); both have been deleted solely or in tandem (Moon et al. 2023b; Wiechmann et al. 2020). There is only one known lactate dehydrogenase in *A. woodii*, the electron bifurcating LDH/ETH complex, encoded by *lctBCD* (Awo_c08710 – Awo_c08730) (Poehlein et al. 2012). This enzyme complex is known to be responsible for lactate oxidation during growth of *A. woodii* on lactate (Weghoff et al. 2015). Recently, it has been reported that the *lctBCD* genes were highly expressed in the Δ*metVF* mutant grown on fructose where lactate was formed as a side product (Moon et al. 2023a). Therefore, to verify that a possible lactate formation was indeed catalyzed by LctBCD we genetically deleted the LDH/ETF complex. For the generation of the Δ*hydBA/hdcr/lctBCD* mutant, the suicide plasmid pMTL_84151_LW_dlct was constructed, which contains each 1000 bp of upstream flanking region (UFR) of *lctB* and downstream flanking region (DFR) of *lctD* leaving only the start codon of *lctB* and the stop codon of *lctD* (Fig. 1a). For selection, this plasmid carries the *pyrE* gene from *Eubacterium limosum* (Wiechmann et al. 2020) and the chloramphenicol/thiamphenicol resistance cassette (*catP*) from *Clostridium perfringens* (Werner et al. 1977). The plasmid was integrated into the chromosome of the Δ*hydBA/hdcr* mutant by homologous recombination at one flanking region in the presence of thiamphenicol and subsequently, disintegration was carried out by counter-selection with 5-fluoroorotate. Single colonies were picked on agar plates with fructose + formate as carbon and energy source. PCR experiments with primers binding outside the deleted region revealed that the *lctBCD* genes were successfully deleted (Fig. 1b), and the *lctD* gene could not be amplified with primers binding inside of *lctD* (Fig. 1c). Subsequently, the absence of the *lctBCD* genes in the chromosome was confirmed by DNA sequencing (Sanger et al. 1977).

### Heterolactate fermentation with fructose in the ∆*hydBA/hdcr* double mutant

In a previous study we have found conversion of fructose to molecular hydrogen, formate, ethanol and lactate as end products in a Δ*metVF* mutant of *A. woodii* (Moon et al. 2023a). Here, we aimed to redirect fructose metabolism to lactate. Since ethanol was only produced in very minor amounts, and since *A. woodii* has eleven different alcohol dehydrogenases, it was not attempted to genetically delete ethanol production. Hydrogen was produced in huge amounts (Moon et al. 2023a) and therefore we analyzed whether H_2_ production would be abolished in the ∆*hdcr*, and the Δ*hydBA/hdcr* double mutant. The growth phenotype of these mutants has been described before; in brief, they do not grow on fructose, H_2_ + CO_2_, methanol, or formate (Moon et al. 2023b). Therefore, the mutants were grown on fructose + formate, harvested in the exponential growth phase and we then analyzed the fermentation balance from fructose in resting cells. Since we have seen that high concentrations of sugars stimulated production of a reduced end product, ethanol, under certain conditions (Moon and Müller 2021), we performed the experiments with 60 mM instead of 20 mM fructose.

**Fig. 2:**
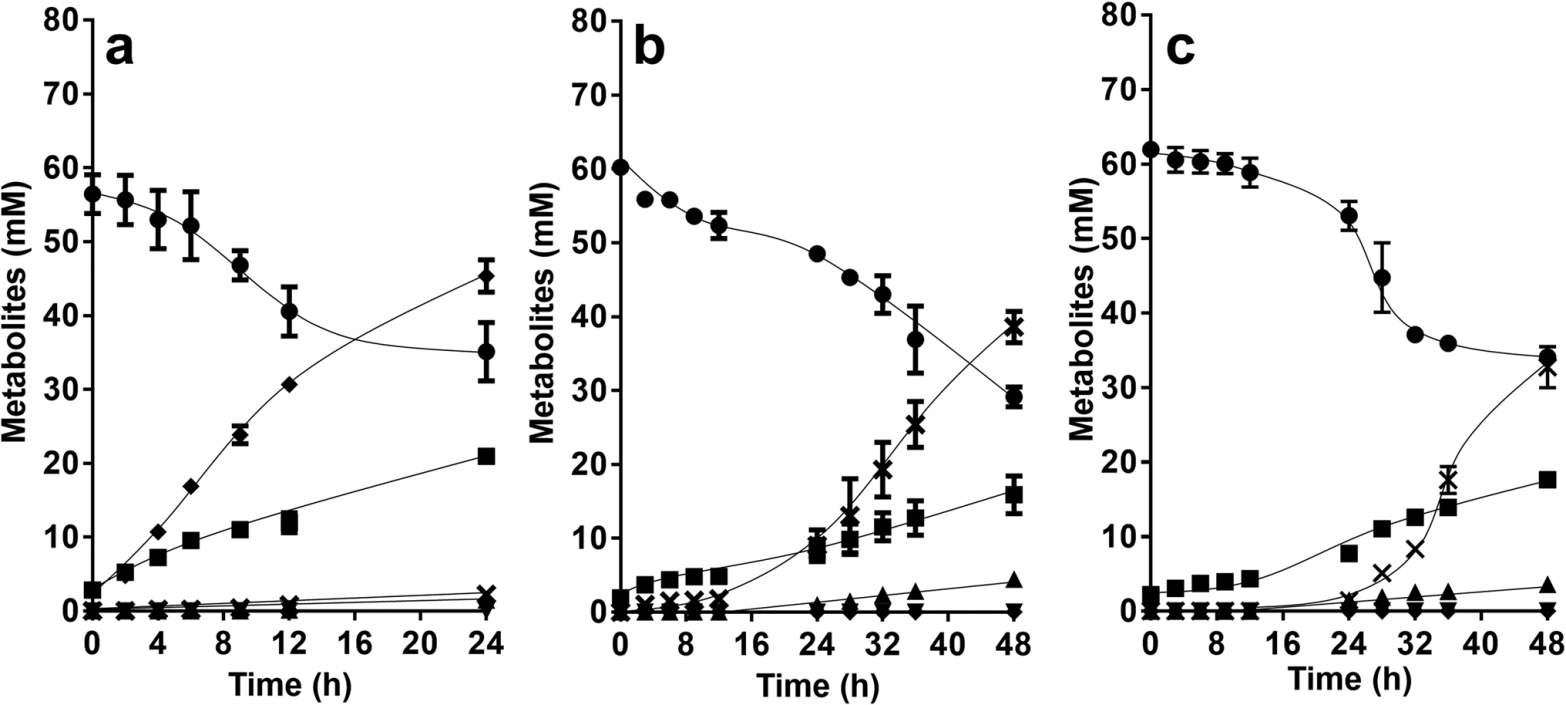
Conversion of fructose in resting cells of *A. woodii*. Cells of the Δ*hdcr* (**a**), Δ*hydBA/hdcr* (**b**) and Δ*hydBA/hdcr/lctBCD* mutants (**c**) were grown in bicarbonate-buffered complex media under a N_2_/CO_2_ atmosphere (80:20, v/v) with 60 mM fructose + 100 mM formate and harvested in the early stationary growth phase. The cell suspensions were prepared in 10 ml of cell suspension buffer (50 mM imidazole, 20 mM MgSO_4_, 20 mM KCl, 20 mM NaCl, 60 mM KHCO_3_, pH 7.0) in 120 ml serum flasks under a N_2_/CO_2_ atmosphere at a final protein concentration of 2 mg/ml. 60 mM fructose was given to the cell suspensions as carbon and energy source. Fructose (●), acetate (■), ethanol (▲), formate (▼), H_2_ (♦) and lactate ( ×) were determined. Each data point presents a mean with standard deviation (SD); *n* = 2 independent experiments

Upon addition of fructose to resting cells of the ∆*hdcr* mutant, 21.4 ± 1.4 mM fructose was consumed, and 21.0 ± 0.4 mM acetate was produced, giving a fructose:acetate ratio of 1:1 (Fig. 2a). Formate was not produced, as expected. As seen before with the Δ*metVF* mutant (Moon et al. 2023a), hydrogen was still formed in huge amounts (45.4 ± 2.2 mM) with a fructose:H_2_ ratio of 1:2.1. Ethanol (1.5 ± 0.0 mM) and lactate (2.1 ± 0.9 mM) were only formed in very minor amounts. Since electrons were apparently released as hydrogen, we checked the effect of deletion of the hydrogenase HydABC in the ∆*hdcr* background. In resting cells of the ∆*hydBA/hdcr* mutant, hydrogen formation was completely abolished, and less acetate (14.0 ± 2.5 mM) was produced from 31.1 ± 1.1 mM fructose with a fructose:acetate ratio of only 1:0.45 (Fig. 2b). Ethanol formation increased a bit (4.5 ± 0.6 mM) with a fructose:ethanol ratio of 1:0.14 and an acetate:ethanol ratio of 1:0.32. In contrast, lactate production increased dramatically from almost zero to 38.6 ± 2.1 mM, giving a fructose:lactate ratio of 1:1.24 and an acetate:lactate ratio of 1:2.76.

Since the *lctBCD* genes are the only genes annotated to encode a lactate dehydrogenase (Poehlein et al. 2012), we expected a complete loss of lactate formation and increase in ethanol production in the triple mutant ∆*hydBA/hdcr/lctBCD*. However, this was not observed. Lactate production had a longer lag phase of around 8 h, compared to the double mutant, but lactate was then produced with rates and yields similar to the double mutant (Fig. 2c).

### Lactate formation from glycine betaine and carbon monoxide

**Fig. 3:**
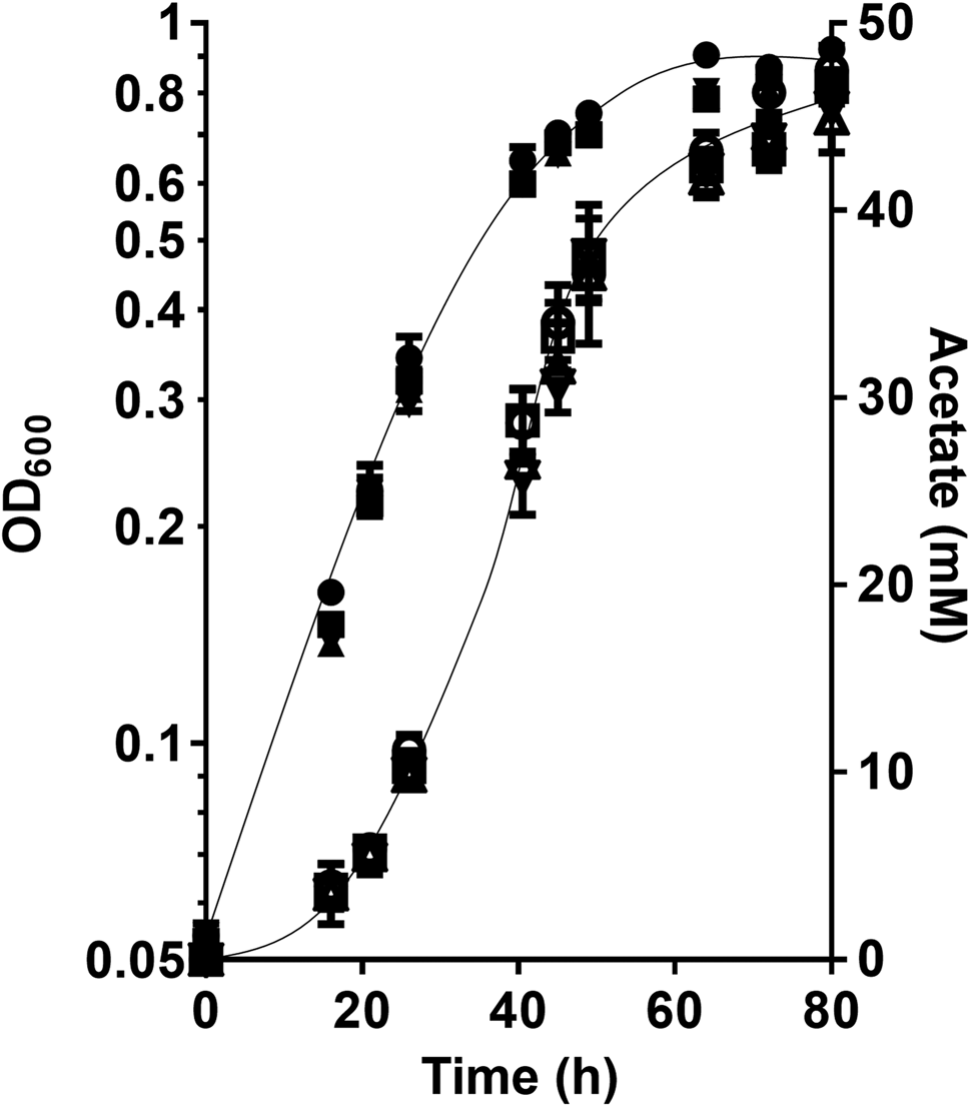
Growth of the *A. woodii* strains on glycine betaine + CO. Growth experiments were performed in 20 ml bicarbonate-buffered complex medium in 120-ml serum flasks with 50 mM glycine betaine under a N_2_/CO_2_/CO atmosphere (72:18:10, v/v/v) at 30 °C. Depicted are the optical densities of the ∆*pyrE* (●), ∆*hdcr* (■), ∆*hydBA/hdcr* (▲), and the ∆*hydBA/hdcr/lctBCD* mutant (▼). Additionally, acetate (open symbols) was determined during growth. Each data point presents a mean ± SD; *n* = 2 independent experiments

**Fig. 4:**
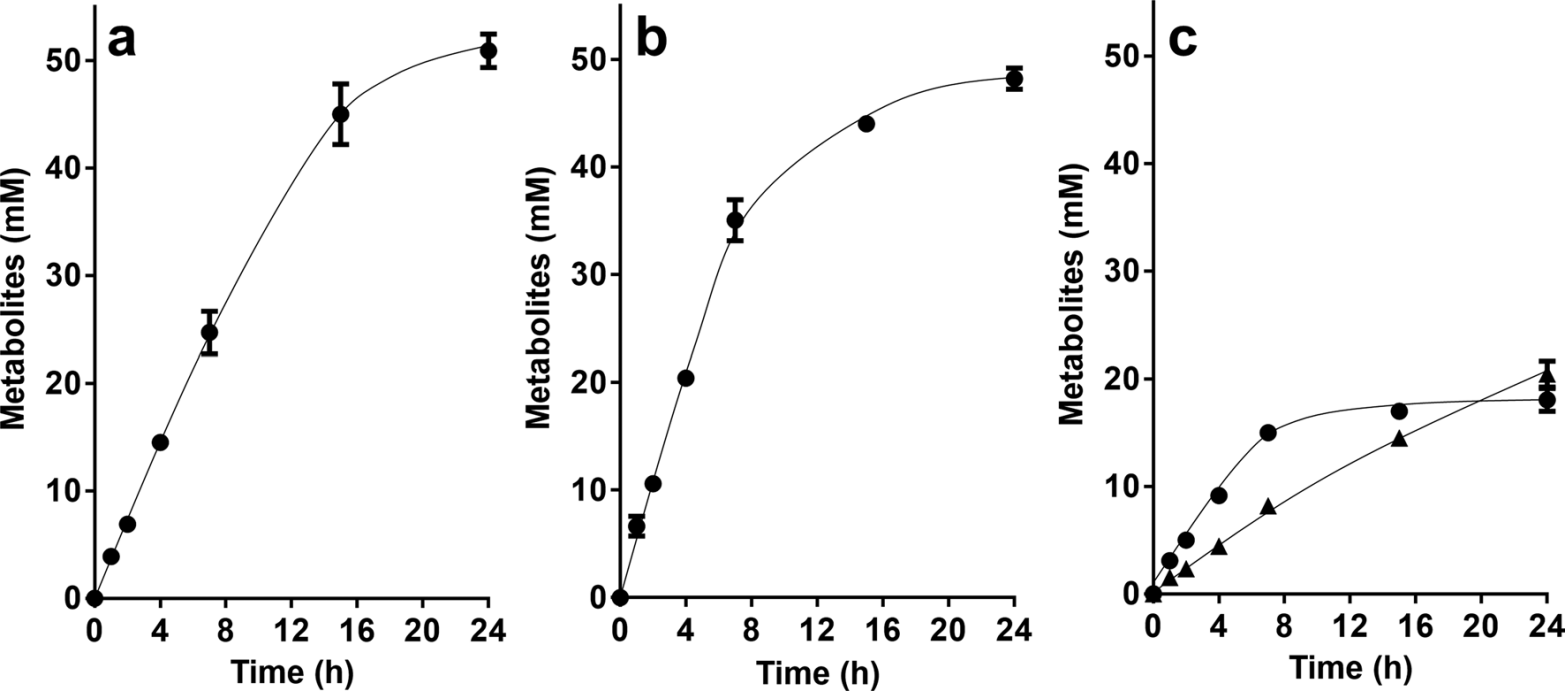
Conversion of glycine betaine + CO in resting cells of *A. woodii*. Cells of the Δ*pyrE* (**a**), Δ*hdcr* (**b**) and Δ*hydBA/hdcr* mutants (**c**) were grown in bicarbonate-buffered complex media under a N_2_/CO_2_/CO atmosphere (72:18:10, v/v/v) with 50 mM glycine betaine and harvested in the early stationary growth phase. The cell suspensions were prepared in 10 ml of cell suspension buffer (50 mM imidazole, 20 mM MgSO_4_, 20 mM KCl, 20 mM NaCl, 60 mM KHCO_3_, pH 7.0) in 120-ml serum flasks with 50 mM glycine betaine under 2 bar of a N_2_/CO_2_/CO (72:18:10, v/v/v) atmosphere at a final protein concentration of 1 mg/ml. Acetate (●) and lactate (▲) were determined. Each data point presents a mean ± SD; *n* = 2 independent experiments

**Fig. 5:**
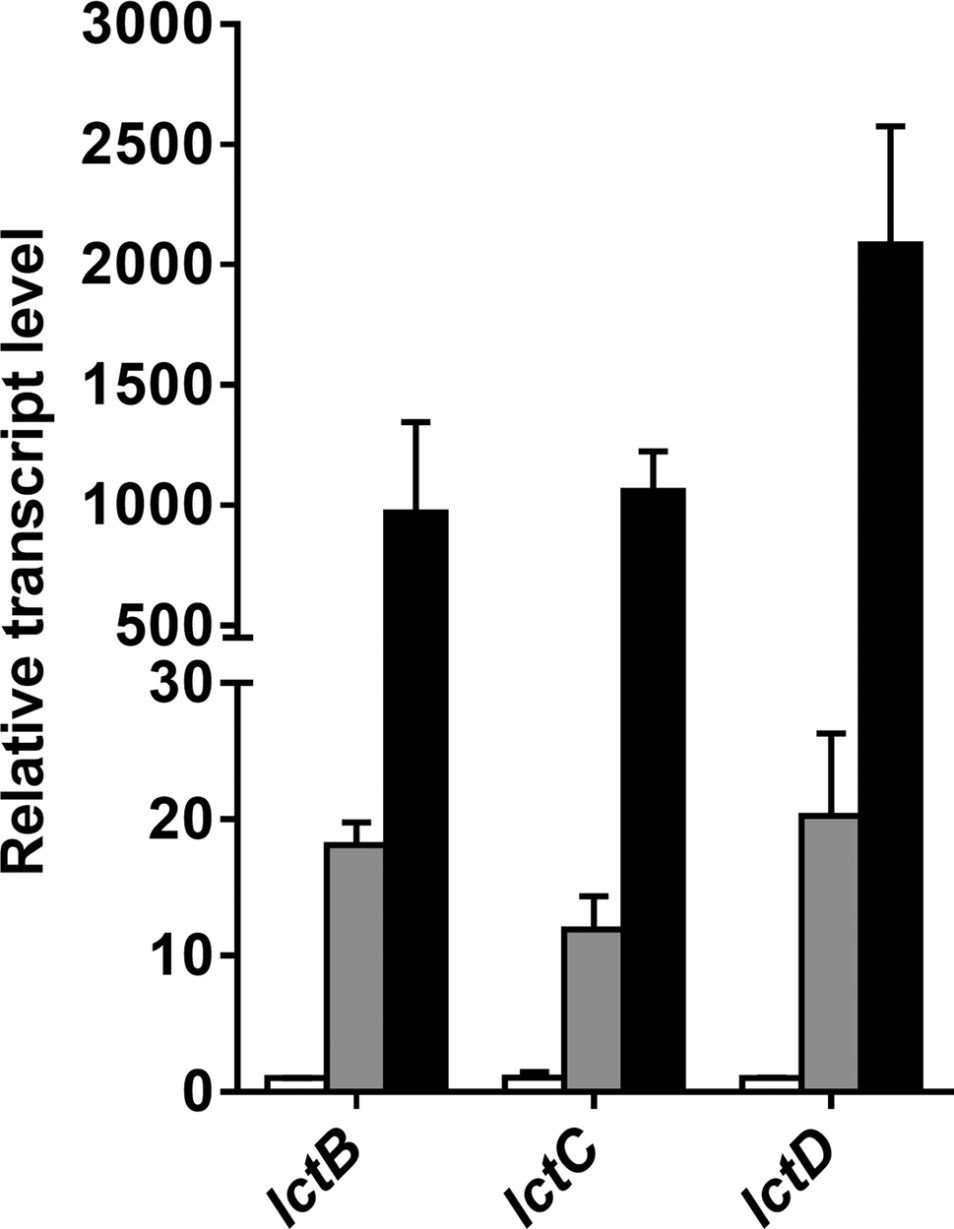
Quantification of transcript levels of the *lctB*, *lctC*, and *lctD* genes in the Δ*hydBA/hdcr* mutant during growth on glycine betaine + CO. cDNA was synthesized from the ∆*pyrE*, Δ*hdcr* and Δ*hydBA/hdcr* mutants grown on 50 mM glycine betaine in bicarbonate-buffered complex media under a N_2_/CO_2_/CO atmosphere (72:18:10, v/v/v). The transcript levels of the *lctB*, *lctC*, and *lctD* genes in the Δ*hdcr* (grey bars) and Δ*hydBA/hdcr* mutants (black bars) were analyzed with quantitative real-time PCR and the relative expression was normalized to a house keeping gene *gyrA*. As control, cDNA of the ∆*pyrE* strain was used (white bars). Each data bar presents a mean ± SD; n = 3 independent experiments

**Fig. 6:**
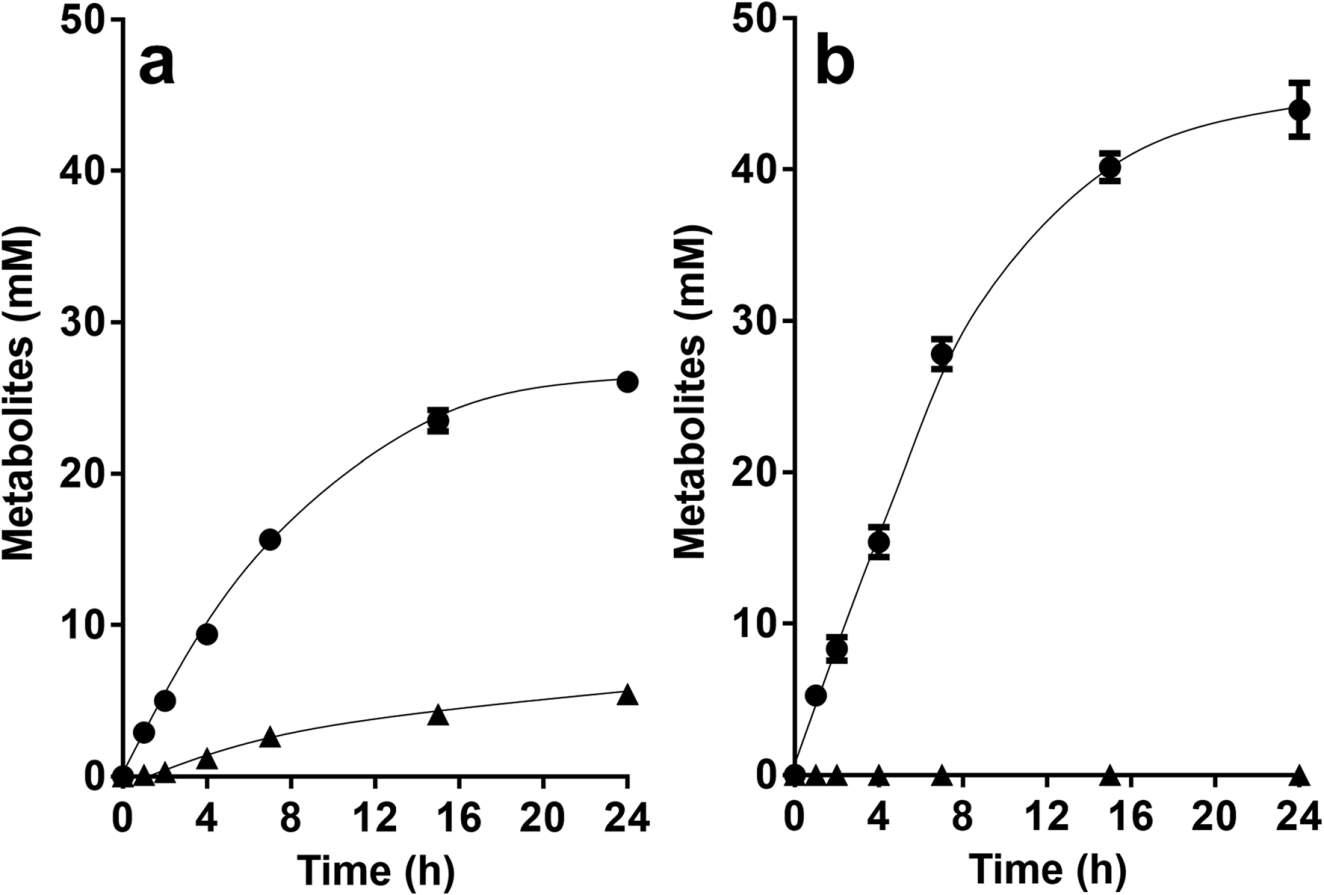
Conversion of glycine betaine + CO in resting cells of the Δ*hydBA/hdcr* mutant under (**a**) CO_2_/HCO_3_^−^- or (**b**) Na^+^-depleted conditions. Cells of the Δ*hydBA/hdcr* mutants were grown in bicarbonate-buffered complex media under a N_2_/CO_2_/CO atmosphere (72:18:10, v/v/v) with 50 mM glycine betaine and harvested in the early stationary growth phase. The cell suspensions were prepared in 10 ml of (**a**) bicarbonate-depleted (50 mM imidazole, 20 mM MgSO_4_, 20 mM KCl, 20 mM NaCl, pH 7.0) or (**b**) Na^+^-depleted cell suspension buffer (50 mM imidazole, 20 mM MgSO_4_, 20 mM KCl, 60 mM KHCO_3_, pH 7.0) in 120-ml serum flasks with 50 mM glycine betaine under 2 bar of a (A) N_2_/CO (90:10, v/v) or (B) N_2_/CO_2_/CO (72:18:10, v/v/v) atmosphere at a final protein concentration of 1 mg/ml. The contaminating Na^+^ concentration was 0.1 mM. Acetate (●) and lactate (▲) were determined. Each data point presents a mean ± SD; *n* = 2 independent experiments

Next, we analyzed whether cells would produce lactate from C1 compounds. The wild type of *A. woodii* was shown to grow on methanol + CO which are converted to acetate; the methyl-group and CO are intermediates of the WLP which are condensed by CODH/ACS to acetyl-CoA (Litty et al. 2022). The HDCR is not involved in that metabolism. Since the ∆*hdcr* and the ∆*hydBA/hdcr* mutants do not grow on methanol (Moon et al. 2023b) regardless of the presence or absence of CO, we tested for growth on another methyl group-containing substrate, glycine betaine, that *A. woodii* can use as carbon and energy source (Lechtenfeld et al. 2018). We recently showed that the ∆*hdcr* and the ∆*hydBA/hdcr* mutants grow on glycine betaine and produce formate as final product alongside acetate (Moon et al. 2023b). Glycine betaine serves as methyl group donor and dimethylglycine is excreted by the cells (Lechtenfeld et al. 2018). The ∆*pyrE* as well as the ∆*hdcr*, ∆*hydBA/hdcr*, ∆*hydBA/hdcr/lctBCD* mutants grew well on 50 mM glycine betaine + CO and produced only acetate (∆*pyrE,* 47.5 ± 1.3 mM; ∆*hdcr,* 46.6 ± 2.1 mM; ∆*hydBA/hdcr,* 44.9 ± 1.8 mM; ∆*hydBA/hdcr/lctBCD,* 45.5 ± 0.6 mM) *via* the WLP similar to growth on methanol + CO (Litty et al. 2022) (Fig. 3). We then checked for product formation in resting cells. Resting cells of the ∆*pyrE* strain produced 50.9 ± 1.6 mM acetate from 50 mM glycine betaine and CO (Fig. 4a) and the same was true for the HDCR mutant (48.2 ± 1.0 mM) (Fig. 4b), as expected. Cells produced hydrogen (0.5 mM in both strains), most likely from CO oxidation. CO oxidation is coupled to reduction of ferredoxin followed by the production of molecular hydrogen in two steps: first, reduced ferredoxin is reoxidized by the Rnf complex (with reduction of NAD) (Hess et al. 2013) and the electron-bifurcating hydrogenase then forms hydrogen from reduced ferredoxin and NADH (Schuchmann and Müller 2012). Therefore, we reasoned that deletion of the electron bifurcating hydrogenase should redirect electrons to another acceptor. Indeed, resting cells of the ∆*hydBA/hdcr* double mutant no longer produced H_2_ but lactate instead, alongside with acetate (Fig. 4c). Acetate production was a bit faster, but final acetate and lactate concentrations were similar. From 50 mM glycine betaine + CO, 18.1 ± 1.1 mM acetate and 20.4 ± 0.5 mM lactate were formed with an acetate:lactate ratio of 1:1.1. As a minor product, we also detected 2.5 mM ethanol. In agreement with the lactate production, we found that the *lctBCD* genes were highly upregulated in the ∆*hydBA/hdcr* mutant during glycine betaine + CO fermentation (Fig. 5). Compared to the ∆*pyrE* strain, the *lctB* gene in the ∆*hydBA/hdcr* mutant was upregulated with a log_2_ fold change of 9.9 ± 0.6. The same was true for the *lctC* gene with a log_2_ fold change of 10.0 ± 0.2 and the *lctD* gene with a log_2_ fold change of 11.0 ± 0.3. Lactate must have been formed from acetyl-CoA *via* carboxylation to pyruvate by pyruvate:ferredoxin oxidoreductase (PFOR), and indeed, a reduced lactate formation was observed under CO_2_/bicarbonate-depleted conditions (Fig. 6a) compared to CO_2_/bicarbonate-rich conditions (cf. Figure 4c). Since NADH is required for lactate production by the LDH/ETF complex, the Rnf complex must be involved i.e., the lactate production must be Na^+^ dependent. Indeed, lactate production (cf. Figure 4c) was completely abolished in the absence of NaCl and the ∆*hydBA/hdcr* mutant produced only acetate (44.0 ± 1.8 mM) (Fig. 6b).

**Fig. 7:**
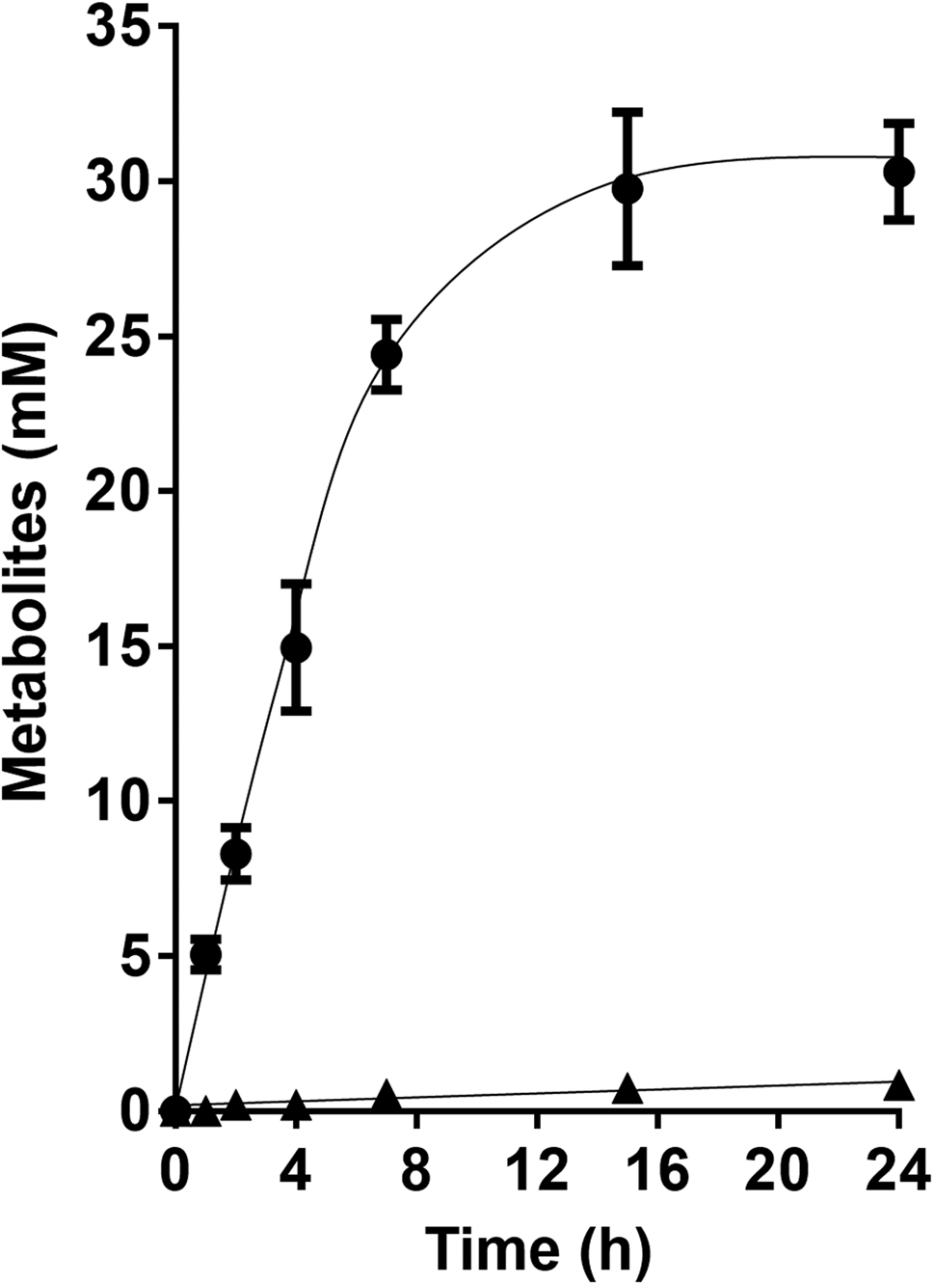
Lactate formation from glycine betaine + CO was abolished in resting cells of the Δ*hydBA/hdcr/lctBCD* mutant. Cells of the Δ*hydBA/hdcr/lctBCD* mutant were grown in bicarbonate-buffered complex media under a N_2_/CO_2_/CO atmosphere (72:18:10, v/v/v) with 50 mM glycine betaine and harvested in the early stationary growth phase. The cell suspensions were prepared in 10 ml of cell suspension buffer (50 mM imidazole, 20 mM MgSO_4_, 20 mM KCl, 20 mM NaCl, 60 mM KHCO_3_, pH 7.0) in 120-ml serum flasks with 50 mM glycine betaine under 2 bar of a N_2_/CO_2_/CO (72:18:10, v/v/v) atmosphere at a final protein concentration of 1 mg/ml. Acetate (●) and lactate (▲) were determined. Each data point presents a mean ± SD; *n* = 2 independent experiments

In the ∆*hydBA/hdcr/lctBCD* triple mutant, lactate formation was nearly completely abolished (Fig. 7), demonstrating that lactate is produced by the electron bifurcating LDH/ETF complex. Interestingly, the Δ*hydBA/hdcr/lctBCD* mutant produced double the amount of ethanol (6.0 ± 0.2 mM) compared to the Δ*hydBA/hdcr* mutant, indicating electrons are partially shifted towards ethanol production in the absence of the LDH/ETF complex.

## Discussion

Acetogenic bacteria are prime candidates as biocatalysts required to transform our bioeconomy to a sustainable, sugar-free bioeconomy. This group of bacteria does not require oxygen, is easy to handle under strict anoxic conditions, grows robust even in industrial size fermenters, and can use carbon monoxide (Diekert and Thauer 1978; Diender et al. 2015; Genthner and Bryant 1982; Savage et al. 1987; Weghoff and Müller 2016), or more reduced C1 compounds such as formate (Moon et al. 2021) or methyl groups derived from various methyl group donors such as methanol or glycine betaine as building blocks for acetyl-CoA (Kremp and Müller 2021; Kremp et al. 2018; Lechtenfeld et al. 2018; Litty et al. 2022; van der Meijden et al. 1984). Electrons for the reduction can be derived from the oxidation of molecular hydrogen, carbon monoxide or organic substrates such as sugars. Moreover, many acetogens can grow mixotrophically on sugars and molecular hydrogen thus increasing the potential for a zero carbon-emission technology (Schuchmann and Müller 2016).

While acetate is the main product for all acetogens, some can naturally produce reduced end products such as ethanol from C1 compounds (Abrini et al. 1994; Köpke et al. 2010; Wilkins and Atiyeh 2011). Production of lactate has rarely been observed from C1 compounds. Lactate is a compound of significant industrial value due to its role as the precursor of PLA (Ahmad et al. 2022). *A. woodii* is one of the best studied acetogens and not only the biochemistry and bioenergetics of the WLP has been studied to a great detail, but also the metabolic pathways that feed C1 substrates into the WLP such as methanol, glycine betaine or CO (Kremp and Müller 2021; Schuchmann and Müller 2014). Recently, we have shown that a methylene-THF reductase deletion mutant performed mixed acid fermentation and produced lactate along with other fermentation products (Moon et al. 2023a). Here, we further investigated lactate production using genetically engineered strains, the ∆*hdcr* and ∆*hydBA/hdcr* mutants. When the electron bifurcating hydrogenase was deleted, lactate was the main product of fructose fermentation, implying that the electrons generated during glycolysis were used for lactate production. Unexpectedly, the ∆*hydBA/hdcr/lctBCD* mutant still produced lactate, although no other *ldh* genes could be identified in the genome. Interestingly, in some microbes NAD^+^-dependent LDH requires fructose-1,6-bisphosphate, an intermediate of the glycolysis, for catalytic activity (Arai et al. 2002; Brown and Wittenberger 1972; Freier and Gottschalk 1987; Machida et al. 1985a, b). In the triple mutant, fructose-1,6-biphosphate could have been accumulated due to slow fructose conversion and triggered the formation/activation of an alternative unknown LDH. But there is also an alternative way to produce lactate during fructose fermentation. An intermediate of glycolysis, dihydroxyacetone phosphate (DHAP) can be converted to methylglyoxal and further reduced to lactaldehyde. Then, lactaldehyde can be reoxidized to lactate (Bhowal et al. 2020; Stewart et al. 2013). The genome of *A. woodii* encodes enzymes that may catalyze these reactions (Poehlein et al. 2012). However, this way does not reoxidize reducing equivalents formed by glycolysis. How exactly lactate is produced from fructose by the double mutant must be investigated by further mutant analyses. Noteworthy, deletion of the LDH/ETF complex abolished lactate formation from C1 compounds (see below), indicating the need for (partial) glycolysis to trigger the alternative LDH way.

Production of lactate from C1 compounds is most attractive for biotechnological applications. Recently, a *lctBCD* deletion mutant of *A. woodii* harboring a lactate dehydrogenase gene from *Leuconostoc mesenteroides* fused to fluorescence-activating and absorption-shifting tag protein (FAST) was shown to produce lactate from H_2_ + CO_2_ (Mook et al. 2022). This strain produced 18.8 mM lactate from H_2_ + CO_2_ in batch experiments, but lactate was a side product with a lactate/acetate ratio of 0.33 (Mook et al. 2022). For exploring lactate production from more reduced C1 compounds, we chose glycine betaine as a methyl group donor plus CO as substrate. As described before for methanol plus CO (Litty et al. 2022), resting cells of ∆*pyrE* strain produced only acetate from glycine betaine + CO according to:

1 $$\mathrm{glycine betaine }+\mathrm{ CO }\to \mathrm{ acetate }+\mathrm{ dimethylglycine }+\mathrm{ ATP}$$

**Fig. 8:**
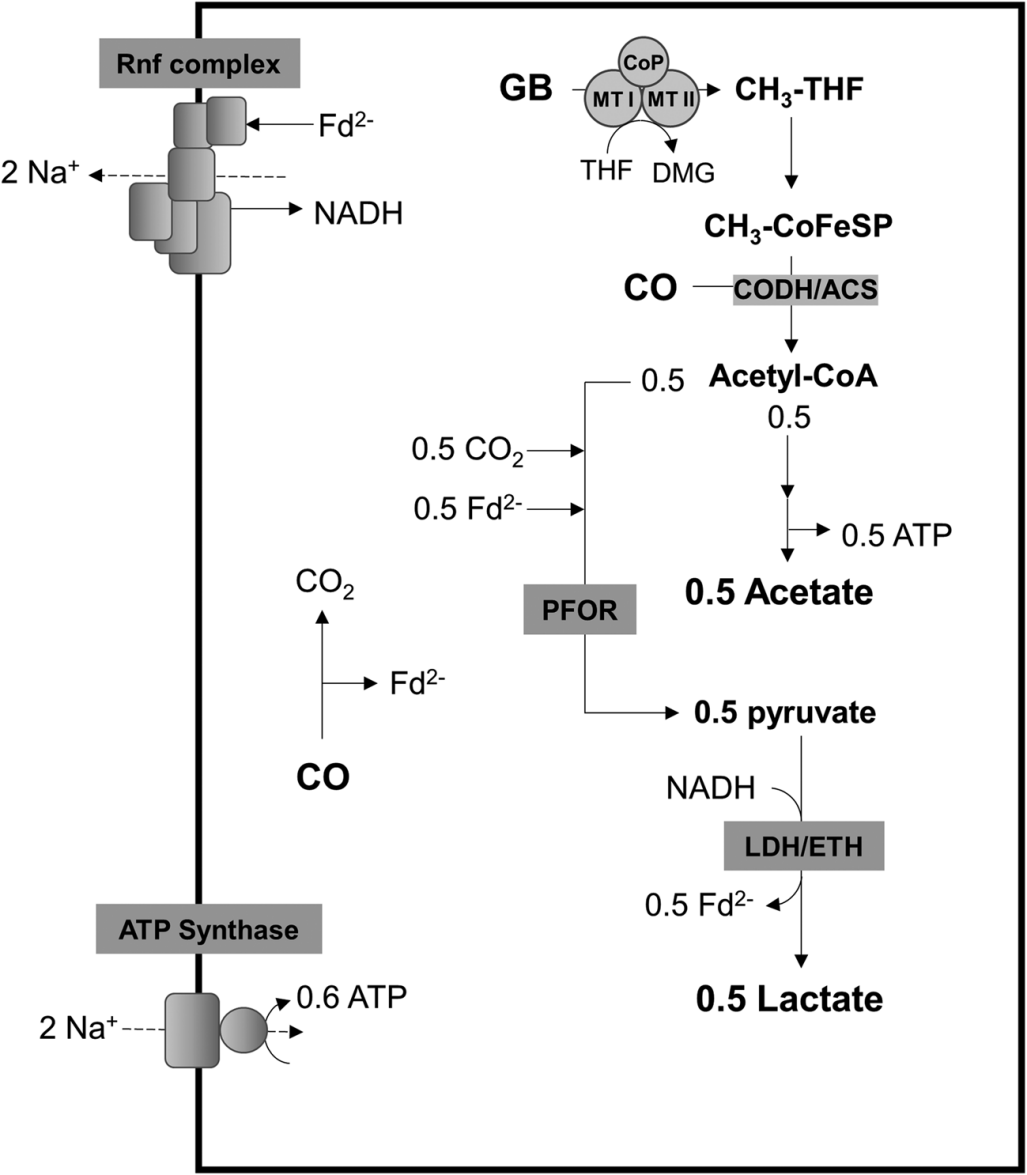
Biochemistry and bioenergetics of lactate production from glycine betaine + CO in the Δ*hydBA/hdcr* mutant of *A. woodii*. Fd, ferredoxin; PFOR, pyruvate:ferredoxin oxidoreductase; LDH/ETF, electron-bifurcating lactate dehydrogenase, GB, glycine betaine; DMG, dimethylglycine; THF, tetrahydrofolate; CODH/ACS, CO dehydrogenase/acetyl-coenzyme A synthase; CoFeSP, corrinoid iron-sulfur protein; MTI, methyltransferase I; MTII, methyltransferase II; CoP, corrinoid protein. The stoichiometry of the ATP synthase is 3.3 Na^+^/ATP (Matthies et al. 2014) and for the Rnf complex a stoichiometry of 2 Na^+^/2 e^−^ is assumed

A likely scenario for lactate formation from glycine betaine + CO in the ∆*hydBA*/*hdcr* mutant is depicted in Fig. 8. The methyl group of glycine betaine is first transferred to THF by the methyltransferase system, yielding methyl-THF which is then condensed with CO and CoA on the CODH/ACS complex for acetyl-CoA production; 0.5 mol acetyl-CoA are then converted to acetate yielding 0.5 mol acetate. The other 0.5 mol of acetyl-CoA have to be reduced to 0.5 mol pyruvate *via* PFOR and the required reduced ferredoxin and CO_2_ were generated from oxidation of CO by the CODH. To produce 0.5 mol lactate, one mol NADH should be required which is produced by the Rnf complex. In sum, 0.5 mol acetate and 0.5 mol lactate are produced from one mol glycine betaine and 2 mol CO according to Eq. 2:

2 $$\mathrm{glycine betaine }+ 2\mathrm{ CO }\to 0.5\mathrm{ acetate }+ 0.5\mathrm{ lactate }+ 0.5 {\mathrm{CO}}_{2} +\mathrm{ dimethylglycine }+ 1.1\mathrm{ ATP}$$

During growth on glycine betaine + CO, the ∆*hydBA*/*hdcr* mutant produced only acetate, similar to the ∆*pyrE* and ∆*hdcr* mutants; the ATP gain of this fermentation is 0.5 mol per mol of carbon of products or educts. On the other hand, during heterolactate fermentation, the ATP gain decreased to 0.37 mol per carbon of products or educts. Therefore, acetogenesis appears to be more favorable over heterolactate fermentation during growth but in resting cells, where a maximum ATP gain is not required, lactate fermentation is obviously preferred for unknown reasons. Moreover, pyruvate produced during growth is probably not accumulated, instead, utilized to build up biomass.

In conclusion, this study shows that a directed genetic engineering of a homoacetogen leads to lactate formation not only from sugar fermentation but also from C1 compounds, which gives a new perspective for industrial applications.

## Data Availability

All datasets and material generated or analyzed in this study are available from the corresponding author upon reasonable request.
